# Factors predicting survival in patients with locally advanced pancreatic cancer undergoing pancreatectomy with arterial resection

**DOI:** 10.1007/s13304-020-00883-7

**Published:** 2020-09-25

**Authors:** Niccolò Napoli, Emanuele Kauffmann, Concetta Cacace, Francesca Menonna, Davide Caramella, Carla Cappelli, Daniela Campani, Andrea Cacciato Insilla, Enrico Vasile, Caterina Vivaldi, Lorenzo Fornaro, Gabriella Amorese, Fabio Vistoli, Ugo Boggi

**Affiliations:** 1 Division of Radiology, Azienda Ospedaliero Universitaria Pisana, University of Pisa, Pisa, Italy; 2 Division of Pathology, Azienda Ospedaliero Universitaria Pisana, University of Pisa, Pisa, Italy; 3 Division of Oncology, Azienda Ospedaliero Universitaria Pisana, University of Pisa, Pisa, Italy; 4 Division of Anesthesia and Intensive Care, Azienda Ospedaliero Universitaria Pisana, University of Pisa, Pisa, Italy; 5 Division of General and Transplant Surgery, Azienda Ospedaliero Universitaria Pisana, University of Pisa, Pisa, Italy

**Keywords:** Locally advanced pancreatic cancer, Pancreatic cancer, Pancreatectomy, Arterial resection, Vascular resection, Prognostic score

## Abstract

Pancreatectomy with arterial resection is a treatment option in selected patients with locally advanced pancreatic cancer. This study aimed to identify factors predicting cancer-specific survival in this patient population. A single-Institution prospective database was used. Pre-operative prognostic factors were identified and used to develop a prognostic score. Matching with pathologic parameters was used for internal validation. In a patient population with a median Ca 19.9 level of 19.8 U/mL(IQR: 7.1–77), cancer-specific survival was predicted by: metabolic deterioration of diabetes (OR = 0.22, *p* = 0.0012), platelet count (OR = 1.00; *p* = 0.0013), serum level of Ca 15.3 (OR = 1.01, *p* = 0.0018) and Ca 125 (OR = 1.02, *p* = 0.00000137), neutrophils-to-lymphocytes ratio (OR = 1.16; *p* = 0.00015), lymphocytes-to-monocytes ratio (OR = 0.88; *p* = 0.00233), platelets-to-lymphocytes ratio (OR = 0.99; *p* = 0.00118), and FOLFIRINOX neoadjuvant chemotherapy (OR = 0.57; *p* = 0.00144). A prognostic score was developed and three risk groups were identified. Harrell’s C-Index was 0.74. Median cancer-specific survival was 16.0 months (IQR: 12.3–28.2) for the high-risk group, 24.7 months (IQR: 17.6–33.4) for the intermediate-risk group, and 39.0 months (IQR: 22.7–NA) for the low-risk group (*p* = 0.0003). Matching the three risk groups against pathology parameters, N2 rate was 61.9, 42.1, and 23.8% (*p* = 0.04), median value of lymph-node ratio was 0.07 (IQR: 0.05–0.14), 0.04 (IQR:0.02–0.07), and 0.03 (IQR: 0.01–0.04) (*p* = 0.008), and mean value of logarithm odds of positive nodes was − 1.07 ± 0.5, − 1.3 ± 0.4, and − 1.4 ± 0.4 (*p* = 0.03), in the high-risk, intermediate-risk, and low-risk groups, respectively. An online calculator is available at www.survivalcalculator-lapdac-arterialresection.org. The prognostic factors identified in this study predict cancer-specific survival in patients with locally advanced pancreatic cancer and low Ca 19.9 levels undergoing pancreatectomy with arterial resection.

## Introduction

The incidence of pancreatic ductal adenocarcinoma (PDAC) is increasing, possibly making this tumor type the second leading cause of cancer-related mortality by the year 2030 [1].This high mortality is mainly related to the biological aggressiveness of PDAC with early haematogenous dissemination [2]. Indeed, at the time of diagnosis, metastases are already visible in approximately 50% of the patients; while, only 20% of the tumors are deemed operable [3]. In the remaining patients, the tumor is either found inoperable (locally advanced tumor) or at a higher risk of margin positivity (borderline-resectable tumor) [4]. The first group includes patients with tumor invasion or abutment > 180° of the celiac trunk and the superior mesenteric artery (SMA), that are classified as stage 3 according to the AJCC [5]. In many of these patients, the superior mesenteric/portal vein is also involved, making surgery extremely complex [6–8]. The overall judgment of unresectability in these patients is mostly based on anticipated high morbidity and mortality [7, 9, 10]not rewarded by an immediately evident survival advantage [6, 9].

Historically, in our institution, vascular involvement has not been deemed an absolute contraindication for resection [6, 11–14].We started with vein resection and, after some experience, we evolved to resect also arterial segments (P-Ar). Initially, arterial resection was considered when technically feasible. After we showed that this approach did not improve survival when compared to palliation [6], we refined our selection criteria to include only patients who had received neoadjuvant chemotherapy [15].The new approach led to improvement in both median survival time and disease-free survival time, despite we were yet unable to predict survival in the individual patient.

Similar results were observed and reported by other groups [7, 8], thus highlighting the need for a careful selection of patients when undertaking such an aggressive surgical approach. While pathology data [16] and molecular tumor profile [17] can be used for prognostic stratification of surgical candidates, most of these data are neither easily achievable nor available before surgery. Actually, when applying currently available selection criteria, including low levels of Ca 19.9 [16–20], long-term survival after P-Ar remains an occasional event that cannot be predicted in the individual patient.

We herein propose the analysis of a consecutive series of patients who received a P-Ar for locally advanced pancreatic cancer (LAPC), aimed to identify pre-operative factors predicting cancer-specific survival (CSS) in this patient population.

## Methods

The Institutional Review Board of the University of Pisa approved this study (study code: LA-PDAC; approval number: 15409_BOGGI). A retrospective analysis of a prospectively maintained database was performed for all patients with a LAPC undergoing P-Ar between August 1, 2006 and December 31, 2018. LAPC was defined as a pancreatic cancer with encasement > 180° of the celiac trunk and/or the superior mesenteric artery (SMA) [5]. All procedures were performed at a single Institution (Division of General and Transplant Surgery, University of Pisa). Exclusion criteria were: metastatic disease, tumor type other than PDAC, arterial resection performed to address only technical issues, and post-operative mortality (defined as any death occurring during the hospital stay or within 90 days from surgery). However, post-operative mortality and morbidity [21–26] were reported to provide a picture of the overall burden of P-Ar.

Data were collected and analyzed according to the Strengthening the Reporting of Observational studies in Epidemiology guidelines for observational studies [27].

### Definition of outcome measures

Time-to-event endpoints were defined according to DATECAN (Definition for Assessment of Time-to-event End-points in CANcer trials) [28]. Namely, overall survival (OS) was defined as the time between the first treatment, either surgery or chemotherapy, and death. Cancer-specific survival (CSS) was defined as the time interval between the first day of treatment, either surgery or chemotherapy, and the day of death related to cancer recurrence. Disease free survival (DFS) was defined as the time interval between the day of surgery and the day of cancer recurrence.

Follow-up time started from the day of the first delivered treatment.

### Selection criteria for surgery

Tumor markers (CEA, Ca 15.3, Ca 125, and Ca 19.9) were assayed in each patient, and all tumors were staged by thoracoabdominal contrast-enhanced computed tomography (CT) scan. Levels of tumor markers used for prognostic determinations were those obtained immediately before surgery. Additional investigations were employed as required in the individual case. Patients with a performance status of 0–1 without evidence of extraregional disease were then discussed in a multidisciplinary tumor board.

In the first part of this experience, when the evidence on neoadjuvant therapies efficacy was lacking or weak, some patients were selected for P-Ar when the tumor appeared to be resectable. Thereafter, P-Ar was considered only after neoadjuvant therapy. In these patients, P-Ar was considered when a decrease in Ca 19.9 levels was noted [18], especially if the drop was ≥ 50% of basal values [19].Radiologic response to neoadjuvant medical therapies was also considered [29]. Although reduction in tumor size was seen as a factor favoring resection, unchanged local tumor status was not considered a contraindication to surgery. Finally, before proceeding to laparotomy, all patients received a staging laparoscopy to rule out occult metastatic disease [30]. Surgery was withheld in patients with proven metastasis.

Patients, and their families, were extensively counseled about the innovative nature of P-Ar. In particular, we disclosed our mortality and morbidity rates, anticipated consequences of the extended resection (such as post-operative diarrhea and diabetes), and the relative inability to define a clear oncologic advantage in the individual patient in comparison with alternative treatments (i.e., continued chemotherapy plus radiotherapy).

### Surgical techniques

P-Ar requires dedicated surgical strategies [6]. In general, dealing with resection and reconstruction of major and vital visceral arteries requires sound knowledge of surgical anatomy of retroperitoneum and mesenteric root, and careful pre-operative planning based on high-quality imaging studies. Background experience with organ procurement, showing unique anatomic views of the retroperitoneum and visceral vessels, and transplantation of abdominal organs, providing the opportunity to practice with vascular reconstructions, were both important for our group.

Briefly, in each patient, surgery begins with a staging laparoscopy and proceeds to laparotomy if no metastasis is discovered. The need for arterial resection is then confirmed by an artery first approach. If no clear plane can be developed between the tumor and the adventitia of the involved artery, and there is opportunity for arterial resection and reconstruction, a P-Ar is performed. As in most patients the venous axis is also involved, vein reconstructability is also carefully considered before embarking upon resection.

For LPAC located in the pancreatic body involving the celiac trunk, a modified Appleby procedure is performed according to the standardized technique that we have reported previously [31]. We do not embolize the hepatic artery pre-operatively. We decide about selective arterial revascularization based on palpation of hepatic arteries in the hepatoduodenal ligament and spectral Doppler waveform, after cross-clamping of the common hepatic artery. Briskness of backward flow from the proximal stump of the common hepatic artery is also considered.

In case of resection of the SMA, total pancreatectomy is preferred over partial pancreatectomy to avoid the consequences of post-operative pancreatic fistula and to have the splenic vessels available for vascular reconstructions. Vascular control, below and above the segments planned for resection, is acquired early on and the specimen is mobilized en bloc with surrounding lympho-neural tissue. In case of venous obstruction, with significant portal hypertension, collateral circulation is spared until the specimen is ready for resection. We do not use routinely a veno-venous by-pass, advocated by other groups [8]. Arteries are often reconstructed using a jump graft, unless only a short segment is resected. We do not use vascular prostheses. Our preferred interposition grafts are the splenic artery (used either as a clockwise rotated segment, when the celiac trunk can be spared, or as a free graft), and the greater saphenous vein. In rare circumstances, we use grafts from deceased donors. In case of gastric ischemia, before partial or total gastrectomy, gastric revascularization is attempted.

Vein reconstruction is performed as described previously [6]. With adequate mobilization of the intestines, the use of jump graft is rarely required, even in case of resection of long vein segments. In simultaneous arterial and venous resections, venous outflow is restored first to reduce congestion of the intestines. Whenever possible, in case of resection of multiple vascular segments, a staged reconstruction technique is used aiming to reduce the time of absolute ischemia of abdominal organs [6].

### Pathology of resected specimens

Two pathologists (D.C., and A.C.I.) reviewed the slides of each case to confirm the diagnosis of PDAC. Slides were also reviewed, if needed, to redefine surgical resection margins according to the 1-mm rule [32].For patients undergoing surgery before, January 1st, 2008, the following margins were examined: pancreas, bile duct, stomach/duodenum, and vascular bed (for vessels not resected en bloc with the specimen). Thereafter, a prospective assessment of the circumferential margin was added.

### Study design and statistical analysis

Categorical variables are summarized as frequencies, percentages and rates. Continuous variables are expressed as mean ± SD, if normally distributed, or as median and interquartile range (IQR). Normality distribution was checked by the Shapiro–Wilk test.

Time-to-event endpoints (OS, CSS, and DFS) were estimated using the non-parametric Kaplan–Meier method.

CSS was chosen as the time-to-event endpoint to be used to define the prognostic factors and to develop the prognostic score.

Missing data were replaced using the multiple imputation method (Multivariate Imputations by Chained Equations—MICE algorithm). Pre-operative covariates having prognostic relevance were identified by univariate Cox proportional-hazard regression. Covariates with a *p*-value < 0.10 were introduced in a multivariate Cox proportional-hazard model to identify a full prognostic model. Redundancy analysis and multi-collinearity were tested using *varclus *with similarity as Spearman and *redun* functions in *R Hmisc* package [33]. The Akaike Information Criterion step function was used to obtain the final reduced model. The final model was tested for Cox proportional-hazard assumptions and was internally validated and calibrated. The concordance value (Harrell’s C-index) was also reported for predicting accuracy of the final model.

Starting from this model, we developed the linear predictor of the regression formula to calculate a score reflecting the individual probability of CSS for each patient. The distribution of the score was divided into tertiles to stratify the overall population into three different risk categories based on anticipated probability of survival: low risk, intermediate risk, and high risk. Differences in median survival time and mortality rates at 12, 24 and 36 months in the risk groups were estimated using Tarone–Ware test and Cochran–Armitage test for trend, respectively. Finally, risk categories were matched to pathological parameters shown to predict survival in the current series of P-Ar.

All statistical analyses were carried out with JMP® 9.0.1 software package for Mac, Copyright© SAS Institute Inc., SAS campus Drive, Cary, NC, USA and R Package, R Core Team (2014): A language and Environment for Statistical Computing, R Foundation for Statistical Computing, Vienna AT using *mice*, *survival*, *rms* and *Hmisc* as packages.

## Results

During the study period, a vascular resection was required in 355 of 1809 patients undergoing pancreatectomy (19.6%). P-Ar was performed in 105 patients (5.8%), including 16 isolated arterial resections (0.8%) and 89 combined arterial and venous resections (4.9%).

PDAC was diagnosed in 80 of 105 patients who received P-Ar (76.1%). In the remaining 25 patients, the following tumor types were identified: malignant intraductal papillary mucinous neoplasm (*n* = 10; 9.5%), distal common bile duct carcinoma (*n* = 5; 4.8%), gallbladder carcinoma (*n* = 1; 0.9%), pancreatic neuroendocrine cancer (*n* = 1; 0.9%), duodenal adenocarcinoma (*n* = 1; 0.9%), adenocarcinoma from unknown primary site (*n* = 1; 0.9%), Ewing sarcoma (*n* = 1; 0.9%), pancreatic metastasis from colorectal carcinoma (*n* = 1; 0.9%), Hodgkin lymphoma (*n* = 1; 0.9%), sclerosing epithelioid fibrosarcoma (*n* = 1; 0.9%),mucinous cystoadenocarcinoma (*n* = 1; 0.9%), and ampullary carcinoma (*n* = 1; 0.9%).

Small liver metastases were present in 2 of 80 PDAC patients (2.5%), but were initially missed due to false-negative result of frozen section histology. In one additional patient, P-Ar was required because of an arterial injury. When these patients were excluded, 77 patients remained with a LAPC who received a planned P-Ar in the absence of obvious metastatic disease and with a low pre-operative level of Ca 19.9. Baseline characteristics of these patients, pathology of resected specimens, details on surgical procedures, and post-operative outcomes are presented in Tables 1, 2, 3, 4. Median Ca 19.9 level was 19.8 U/mL(IQR: 7.1–77.0).

**Table 1:** Baseline characteristics

	Study population
Number of patient	77
Age; mean ± SD; years	62.5 ± 8.6
Female gender	39 (50.6%)
BMI; mean ± SD; Kg/m^2^	23.2 ± 3.2
ASA score; median (IQR)	2 (2–3)
Charlson Comorbidity Index; median (IQR)	5 (4–5)
Comorbidities	
Heart disease	9 (11.7%)
COPD	2 (2.6%)
Hypertension	32 (41.6%)
Diabetes mellitus	22 (28.6%)
Symptoms	
Jaundice	31 (40.3%)
Abdominal pain	46 (59.7%)
Duodenal obstruction	12 (15.6%)
Loss of weight	20 (26%)
Previous abdominal surgery	48 (62.3%)
Laboratory tests	
Leukocytes; median (IQR); n × 10^6^/µL	6.2 (4.6–7.6)
Neutrophils; median (IQR); n × 10^6^/µL	3.4 (2.5–4.5)
Lymphocytes; mean ± SD; n × 10^6^/µL	1.7 ± 0.6
Platelets; median (IQR); n × 10^3^/µL	192 (152–257.5)
Proteins; mean ± DS; g/dL	6.8 ± 0.6
Albumin; mean ± DS; g/dL	3.9 ± 0.5
Ca 19.9; median (IQR); U/mL	19.8 (7.1–77.0)
CEA; median (IQR); KU/L	3.4 (2.1–5.3)
Ca 125; median (IQR); KU/L	12.3 (9.3–21.6)
Ca 15.3; median (IQR); KU/L	20.4 (16.4–27.1)
Neutrophils-to-lymphocytes (NLR); median (IQR)	2.1 (1.4–3.0)
Lymphocytes-to-monocytes (LMR); median (IQR)	3.5 (2.6–4.5)
Platelets-to-lymphocytes ratio (PLR); median (IQR)	113.0 (89.1–172.9)
Albumin-to-globulin ratio; median (IQR)	1.4 (1.2–1.6)
Pre-operative CT scan tumor diameter; median (IQR); mm	30 (25–38)
Tumor location; number (%)	
Head/uncinated process	58 (75.3%)
Body	8 (10.4%)
Tail	3 (3.9%)
Diffuse	8 (10.4%)
Pre-operative tissue diagnosis; number (%)	54 (70.1%)
Arterial involvement (patients) based on imaging findings; number (%)	
Hepatic artery/Celiac trunk alone	36 (46.8%)
Superior mesenteric artery alone	27 (35.1%)
Hepatic artery/celiac trunk + superior mesenteric artery	14 (18.2%)
Pre-operative chemotherapy; number (%)	54 (70.1%)
FOLFIRINOX	30 (39.0%)
Gemcitabine-based	21 (27.3%)
Sequential treatments with different regimens	3 (3.9%)
Number of pre-operative chemotherapy cycles; mean ± SD	7.9 ± 3.3
Duration of chemotherapy; mean ± SD; months	7.1 ± 2.6
Pre-operative radiation therapy; number (%)	4 (5.2%)
Radiologic response to pre-operative medical therapies; number (%)	54 (70.1%)
Stable disease	29 (37.7%)
Response	25 (32.5%)
Postoperative chemotherapy; number (%)	41 (73.2%)
FOLFIRINOX	6 (7.8%)
Gemcitabine-based	35 (45.5%)

*BMI* body mass index, *ASA* American society of anesthesiologists, *COPD* chronic obstructive pulmonary disease, *IQR* interquartile range, *SD* standard deviation

**Table 2:** Pathology of resected tumors

Tumor size; median (IQR); cm	3.5 (3–4)
T status	
T0	1 (1.3%)
T1	4 (5.2%)
T2	37 (48.1%)
T3	11 (14.3%)
T4	24 (31.2%)
N status	
N0	8 (10.4%)
N1	34 (44.2%)
N2	35 (45.5%)
Examined lymph nodes; mean ± SD	75.7 ± 29.2
Metastatic lymph nodes; median (IQR)	3 (2–7)
Number of metastatic lymph nodes	
0	8 (10.4%)
1	7 (9.1%)
2–3	27 (35.1%)
4–7	21 (27.3%)
≥ 8	14 (18.2%)
Lymph node ratio; median (IQR)	0.05 (0.02–0.08)
LODDS; mean ± SD	− 1.2 ± 0.5
R1	16 (20.8%)
Number of positive margins; median (IQR)	1 (1–1)
Perineural Infiltration	66 (85.7%)
Arterial segments with confirmed infiltration Vein	57 (74.0%)
Superior Mesenteric Vein/Portal Vein	49 (68.1%)
Artery*	
Superior Mesenteric Artery	10 (37%)
Celiac Trunk/Hepatic Artery	16 (41%)
Superior Mesenteric Artery + Celiac Trunk/Hepatic Artery	2 (18.2%)

*IQR* interquartile range, *SD* standard deviation, *LODDS* Log (metastatic nodes/non metastatic nodes)

*Two patients had simultaneous infiltration of celiac trunk and superior mesenteric artery

**Table 3:** Surgical procedures in 77 P-Ar for LAPC

	Overall population
Type of pancreatic resection	
Total pancreatectomy	65 (84.4%)
Distal pancreatectomy	8 (10.4%)
Pancreaticoduodenectomy	4 (5.2%)
Surgical approach	
Open	76 (98.7%)
Robotic	1 (1.3%)
Type of vascular resection	
Combined arterial and venous	72 (93.5%)
Isolated arterial	5 (6.5%)
Resected vessel	
Superior mesenteric vein/Portal vein	72 (93.5%)
Inferior vena cava	4 (5.2%)
Superior mesenteric artery alone	27 (35.1%)
Celiac trunk/Hepatic artery alone	39 (50.6%)
Superior mesenteric artery and Celiac trunk/Hepatic artery	11 (14.3%)
Type of vascular reconstruction (understood as exclusive)	
No reconstruction	4 (5.2%)
Venorrhaphyor patch closure	0 (0%)
Direct end-to-end anastomosis	17 (22.1%)
Interposition graft	11 (14.3%)
Combination thereof	45 (58.4%)
Multiviseral resection	28 (36.4%)
Gastric resection	20 (26.0%)
Colic resection	4 (5.2%)
Management of the stomach	
Pylorus preservation	31 (40.3%)
Resection pyloric ring/gastric antrum	23 (29.9%)
Total gastrectomy	16 (20.8%)
Operative time; mean ± SD; min	605.9 ± 134.1

*SD* standard deviation

**Table 4:** Post-operative results

	Overall population
Length of stay; median (IQR); days	24 (17.5–30)
Patients receiving blood transfusions; number (%)	22 (28.6%)
Units transfused per patient; median (IQR); number	2 (2–3.3)
Post-operative complications; number (%)	58 (75.4%)
Grade 1	6 (7.8%)
Grade 2	30 (39.0%)
Grade 3a	6 (7.8%)
Grade 3b	3 (3.9%)
Grade 4a	4 (5.2%)
Grade 4b	1 (1.3%)
Grade 5 **^**	8 (10.3%)
Severe post-operative complications (≥ 3a)	22 (28.6%)
Comprehensive Complication Index; median (IQR)	22.6 (4.4–39.5)
Type of post-operative complications	
Post-Pancreatectomy Hemorrhage (PPH)*	21 (27.3%)
PPH grade B	19 (24.7%)
PPH grade C	2 (2.6%)
Delayed Gastric Emptying (DGE)	21 (27.3%)
DGE grade A	6 (7.8%)
DGE grade B	12 (15.6%)
DGE grade C	3 (3.9%)
Enteric Fistula	4 (5.2%)
Vascular/ischemic complications	4 (5.2%)
Medical Complications	38 (49.4%)
Cardiologic	5 (6.5%)
Pneumologic	18 (23.4%)
Repeat surgery at 90 days	7 (9.1%)

*IQR* interquartile range

*Figures for PPH grade A are not reported as no case was recorded

**Fig. 1:**
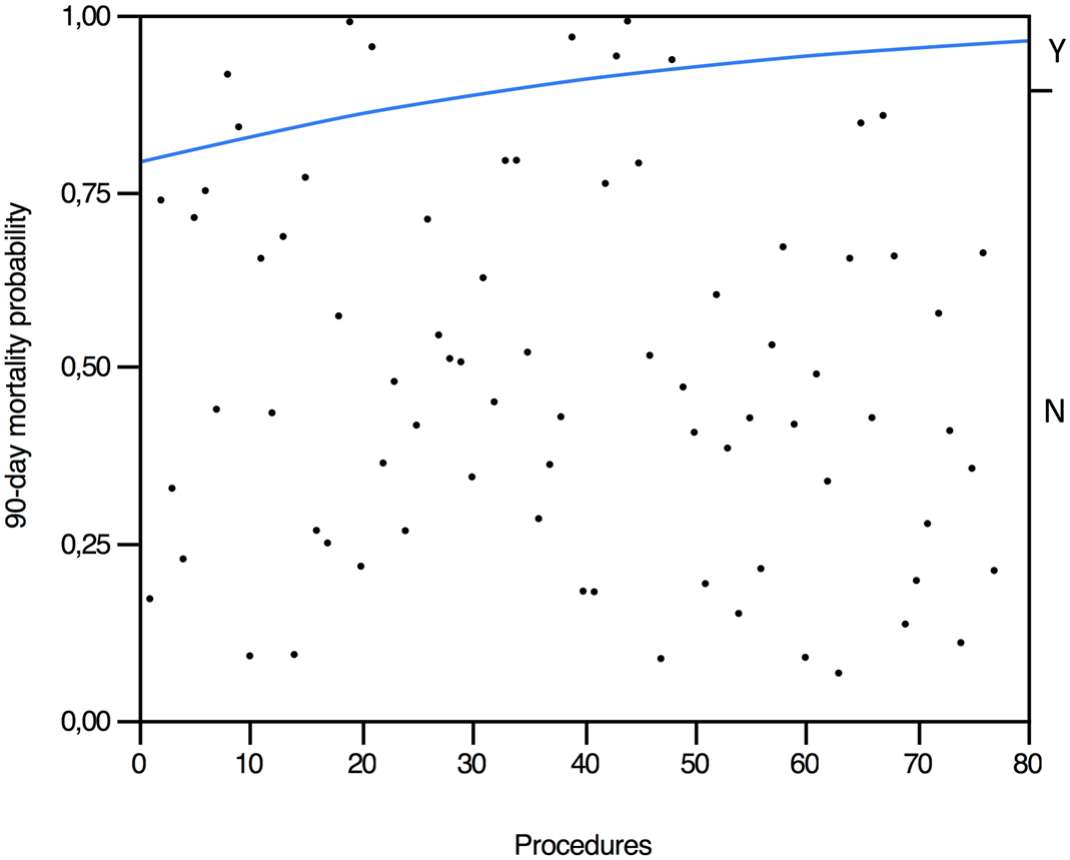
Logistic plot showing a positive relationship between postoperative mortality and number of performed procedures. The blue line is the probability curve for post-operative death. Y: yes (i.e., mortality). N: no (i.e., no mortality).Please note that cases are represented as dots. Eight dots, corresponding to post-operative deaths, are located above the blue line showing the probability of post-operative deaths along the cumulative experience

The overall 90-day post-operative mortality was 10.3% (8/77). An important decline was noted during the study period (2006–2008 2/16: 12.5%) (2009–2011 2/11: 18.1%) (2012–2014 3/20: 15.0%) (2015–2018 1/30: 3.3%). Deaths occurred at procedures number 8th, 9th, 19th, 21st, 39th, 43rd, 44th, and 48th. We recorded no additional deaths in the last 29 consecutive procedures (Fig. 1). Reasons for post-operative deaths were: intestinal ischemia (*n* = 3); intraluminal bleeding (*n* = 2); liver ischemia (*n* = 1); consequences of small bowel perforation (*n* = 1); and myocardial infarction (*n* = 1).

### Study population

Excluding eight post-operative deaths, follow-up data were available for each of the remaining 69 patients who constituted the study population.

Neoadjuvant chemotherapy was administered to 48 patients (69.6%) consisting of either FOLFIRINOX (*n* = 31; 45.6%) or gemcitabine-based schedules (*n* = 19; 27.9%). The mean number of chemotherapy cycles was 7.9 ± 3.3 and the mean duration of treatment was 7.1 ± 2.5 months. Pre-operative chemoradiation was used in three patients.

A total of 47 patients (68.1%) received adjuvant chemotherapy. Twenty-seven patients (39.1%) received both neoadjuvant and adjuvant chemotherapy. Adjuvant chemotherapy was started within 8 weeks from surgery in 15 patients (34.9%). Twenty-six patients (55.3%) completed full course adjuvant chemotherapy.

**Fig. 2:**
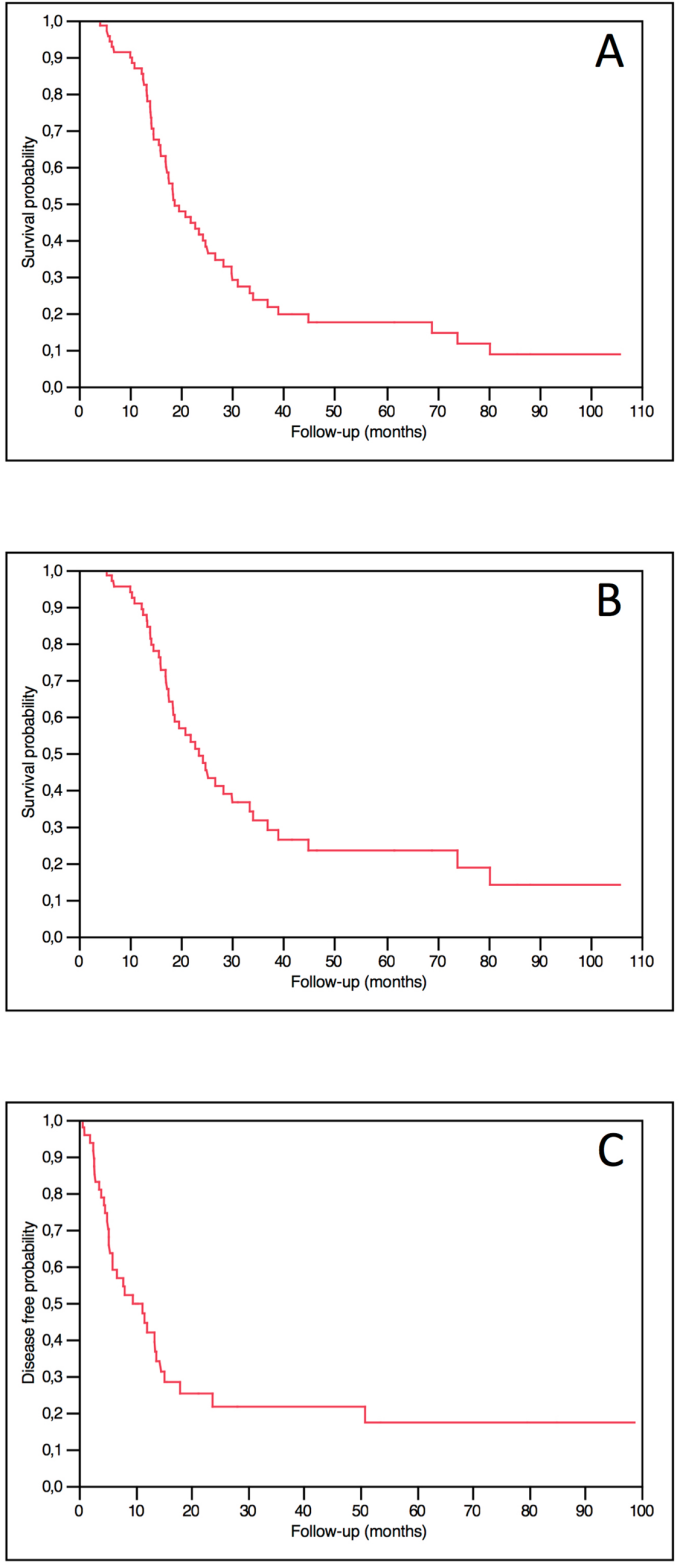
Kaplan–Meier survival curves for OS (**a**), CSS (**b**), and DFS (**c**)

After a median follow-up time of 18.4 months (IQR: 13.7–30.0), median OS was 18.8 months (IQR: 14–34.1), median CSS was 23.5 months (IQR: 16–44.9) and median DFS was 9.6 months (IQR: 4.7–23.8) (Fig. 2a–c).Median follow-up time for censored cases was 23.7 months (IQR: 13.3–34.6).

It may be worth to note that median OS, CSS, and DFS were similar for patients who received resection of the SMA and of the celiac trunk/hepatic artery. In particular, after SMA resection median OS was 18.9 months (IQR: 15.1–74), CCS was 24.6 months (IQR: 17–80.3), and DFS was 9.6 months (IQR: 4.5–NA). Equivalent figures for celiac trunk/hepatic artery were 17.7 months (IQR: 12.6–30), 22.8 months (IQR: 13.9–33.4), and 8.2 months (IQR: 5.2–14.6). None of the differences was statistically relevant.

### Identification of prognostic factors for CSS and development of prognostic score

The rate of missing pre-operative data was 1.29%.

Pre-operative prognostic factors predicting CSS in univariate analysis and multivariate cox regression were metabolic deterioration of diabetes (OR = 0.22, *p* = 0.0012), pre-operative platelet count (OR = 1.00; *p* = 0.0013), pre-operative serum level of Ca 15.3 (OR = 1.01, *p* = 0.0018) and Ca 125 (OR = 1.02, *p* = 0.00000137), pre-operative neutrophils-to-lymphocytes ratio (OR = 1.16; *p* = 0.00015), pre-operative lymphocytes-to-monocytes ratio (OR = 0.88; *p* = 0.00233), pre-operative platelets-to-lymphocytes ratio (OR = 0.99; *p* = 0.00118), and FOLFIRINOX-based neoadjuvant chemotherapy (OR = 0.57; *p* = 0.00144). Of note, the OR of continuous variables, such as tumor markers, applied to each unit of increment. For instance, the OR of Ca 125 was 1.02 for a single unit of increment (e.g., from 12 to 13 U/L), but was 1.02 × 10 (i.e., 10.2) if the level of the marker increased by 10 units (e.g., from 12 to 22 U/L).

**Fig. 3:**
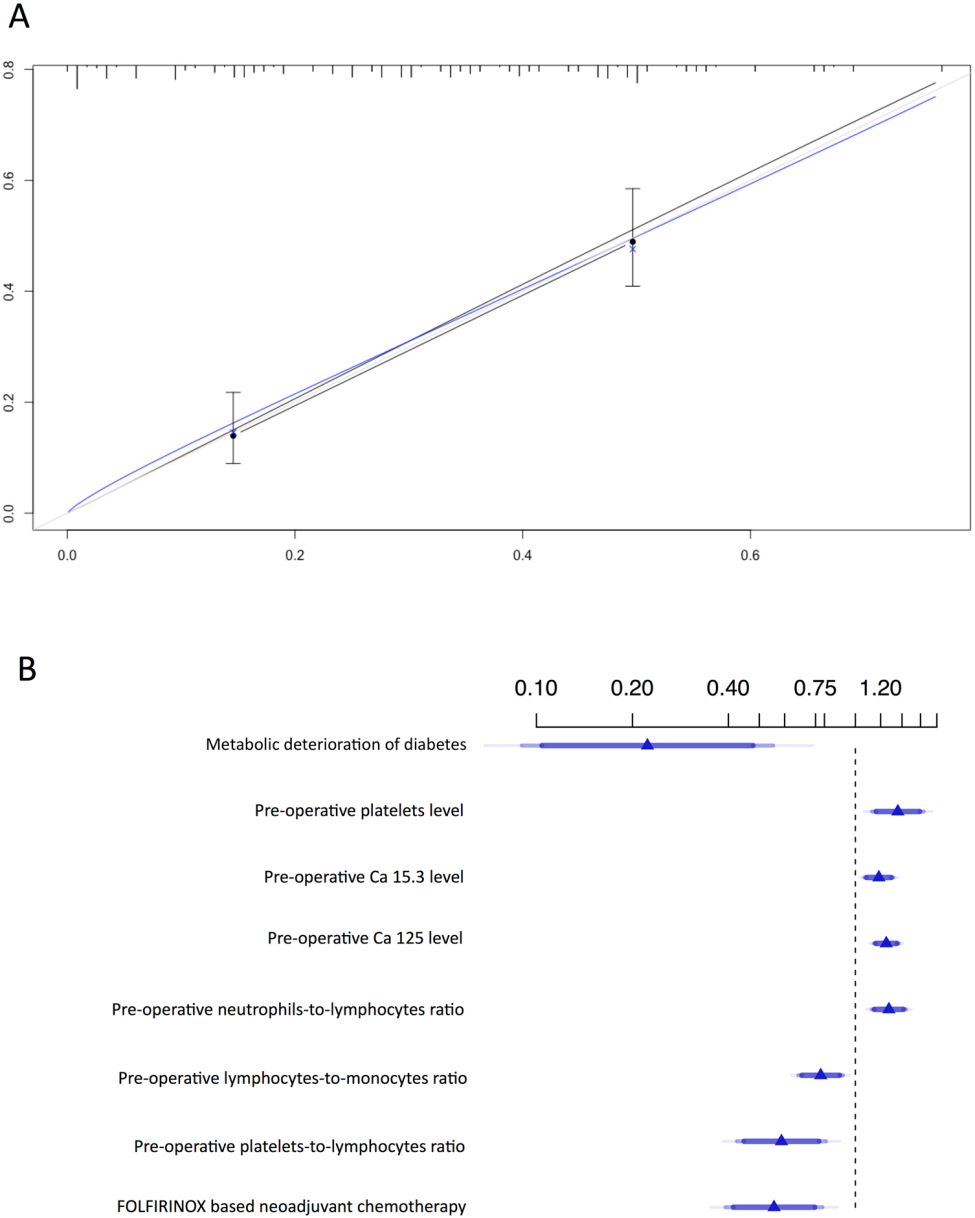
Calibration curve (**a**) and forest plot diagram (**b**) of the prognostic model. In the forest plot, triangles represent the hazard ratio and horizontal bars the lower and upper limits of the 95% confidence interval

Univariate and multivariate analyses and the final model used to develop the prognostic model are reported in Table 5. Concordance value (Harrell’s C-Index) was 0.74 (likelihood ratio = 102.4 *p* < 2^−16^). The calibration curve and the forest plot of the final prognostic model are reported in Fig. 3. An online calculator is available at www.survivalcalculator-lapdac-arterialresection.org.

**Table 5:** Univariate and multivariate Cox proportional hazard regression

	Univariate analysis				Multivariate analysis			
	Beta coefficient	HR	95% CIs	*p*	Beta coefficient	HR	SE	*p*
Age	0.033	1.03	0.10–1.07	0.079				
Male gender	0.028	1.33	0.72–2.46	0.36				
Body mass index	− 0.055	0.95	0.86–1.04	0.27				
Cardiopathy	0.63	1.87	0.78–4.51	0.16				
Chronic obstructive pulmonary disease	1.19	3.29	0.78—13.9	0.11				
Metabolic deterioration of diabetes	−1.652	0.19	0.03–1.41	0.100	−1.503	0.22	0.09–0.55	0.0012
ASA score	0.055	1.06	0.65–1.73	0.83				
Symptomatic	0.49	1.63	0.58–4.58	0.35				
Jaundice	0.26	1.30	0.71–2.39	0.40				
Abdominal pain	0.10	1.11	0.60–2.05	0.74				
Weight loss	− 0.037	0.96	0.47–1.97	0.92				
Previous abdominal surgery	− 0.38	0.69	0.37–1.28	0.24				
Pre-operative leukocytes level	0.112	1.12	1.008–1.28	0.071				
Pre-operative neutrophils level	0.159	1.17	1.03–1.32	0.015				
Pre-operative platelets level	0.0000026	1.0000026	0.99–1.005	0.060	0.00000269	1.00	1.00–1.00	0.0013
Pre-operative Ca 15.3 level	0.0110	1.011	1.002–1.02	0.084	0.01355	1.01	1.005–1.02	0.0018
Pre-operative Ca 19.9 level	0.00008	1.00	0.99–1.00	0.37				
Pre-operative Ca 125 level	0.0165	1.016	1.009–1.04	0.0039	0.01521	1.02	1.009–1.02	0.0000013
Pre-operative neutrophils-to-lymphocytes ratio	0.0954	1.100	1.025–1.19	0.010	0.1509	1.16	1.08–1.25	0.00015
Pre-operative lymphocytes-to-monocytes ratio	− 0.174	0.840	0.70–1.05	0.081	− 0.1224	0.88	0.82–0.96	0.00233
Pre-operative platelets-to-lymphocytes ratio	0.0037	1.003	1.001–1.007	0.013	− 0.00586	0.99	0.99–0.99	0.00118
Pre-operative albumin-to-globulin ratio	0.015	1.02	0.54–1.90	0.96				
Tumor size at CT scan	0.017	1.02	0.99–1.04	0.13				
Pre-operative CT scan SMA involvement	− 0.47	0.63	0.34–1.15	0.13				
Pre-operative CT scan celiac trunk/hepatic artery involvement	− 0.013	0.99	0.54–1.81	0.97				
Tumor location								
Head	− 0.38	0.68	0.33–1.40	0.30				
Body	0.05	1.05	0.50–2.21	0.91				
Tail	0.42	1.51	0.52–4.38	0.44				
Pre-operative biliary drainage	− 0.34	0.71	0.17–2.98	0.64				
Gemcitabine based neoadjuvant chemotherapy	0.09	1.10	0.53–1.99	0.87				
FOLFIRINOX-based neoadjuvant chemotherapy	− 0.6017	0.548	0.313	0.054	− 0.5636	0.57	0.40–0.81	0.00144

### Predictive value of the model on CSS

**Fig. 4:**
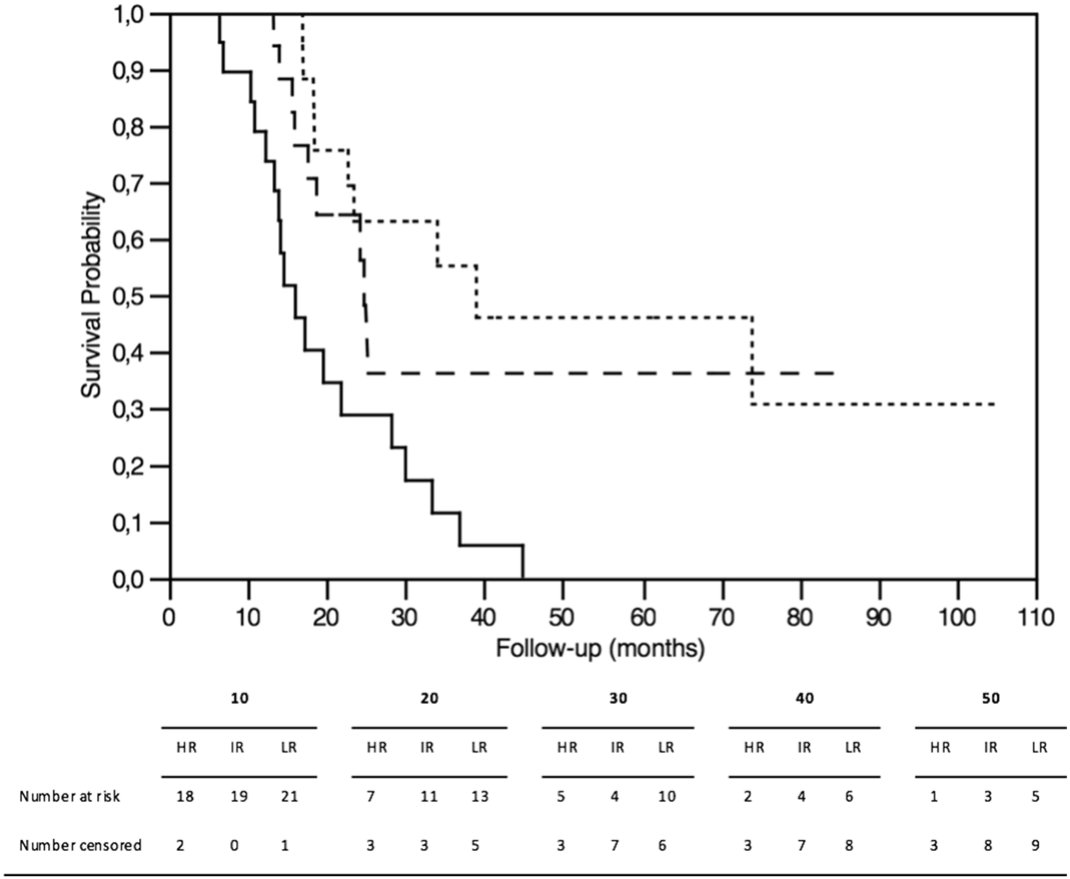
Kaplan–Meier curves for CSS in low-risk (dotted line), intermediate-risk (dashed line), and high-risk (continuous line) groups

The median value of the prognostic score was 165 (IQR: 155–178.5). When the 33.3rd and 66.6th percentiles were used as breakpoints, 21 patients were allocated to the high-risk group (34.4%), 19 patients to the intermediate-risk group (31.1%) and 21 patients to the low-risk group (34.4%). Corresponding median CSS times were 16.0 months (IQR: 12.3–28.2) for the high-risk group, 24.7 months (IQR: 17.6–33.4) for the intermediate-risk group, and 39.0 months (IQR: 22.7–NA) for the low-risk group (*p* = 0.0003). Median OS and DFS were 14.2 months (IQR: 10.8–21.9) and 5.0 months (IQR: 2.7–6.8) in the high-risk group, 24.3 months (IQR: 15.7–69) and 11.7 months (IQR: 8.2—NA) in the intermediate-risk group, and 31.1 months (IQR: 18.5–74) and 14.6 months (IQR: 8.2–NA) in the low-risk group (*p* = 0.0003 and 0.0081, respectively) (Fig. 4).

One year after surgery, mortality due to cancer recurrence was 21.1% in the high-risk group, and 0 in both intermediate-risk and low-risk groups (*p* = 0.01). Equivalent figures at 2 and 3 years were 72.2.8, 42.9, 37.5% (*p* = 0.04) and 88.9, 75.0 and 53.9% (*p* = 0.03), respectively.

### Correlation between risk groups and pathological features

**Fig. 5:**
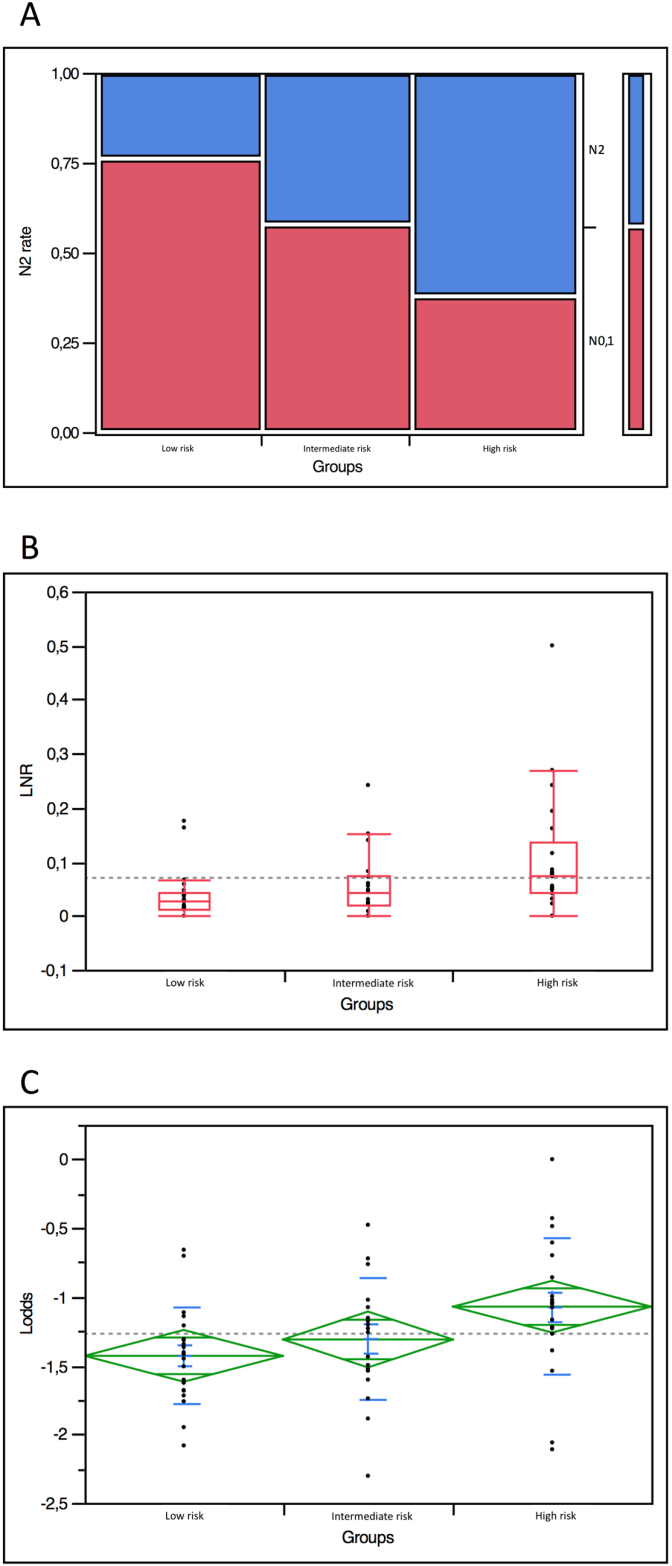
Correlation between risk groups and pathology parameters predicting CSS. **a** Spine plot showing the proportion of N2 (blue) and N0-1 (red) tumors in the three risk groups. **b** Box plot demonstrating median and interquantile range of lymph-node ratio in the three risk groups. Dotted line indicates the median value of the overall population. **c** Diamond plot showing median and standard deviation of logarithm odds of positive nodes. Dotted line indicates the mean value of the overall population

The rate of patients staged N2 was 61.9% in the high-risk group, 42.1% in the intermediate-risk group, and 23.8% in the low-risk group (*p* = 0.04). Similarly, median values of lymph-node ratio and mean values of logarithm odds of positive nodes were 0.07 (IQR: 0.05–0.14), 0.04 (IQR: 0.02–0.07), and 0.03 (IQR: 0.01–0.04) (*p* = 0.008), and − 1.07 ± 0.5, − 1.3 ± 0.4, and − 1.4 ± 0.4 (*p* = 0.03) in the three risk groups, respectively (Fig. 5a–c).

Interestingly, there was no difference in the proportion of T stages across the three risk groups. Namely, T stages were distributed in low-, intermediate- and high-risk groups as follows: T0: 100% vs. 0 vs. 0 (*p* = 0.38); T1: 100% vs. 0 vs. 0 (*p* = 0.38); T2: 42.9% vs. 14.3% vs. 42.9% (*p* = 0.59); T3: 37.8% vs. 29.7% vs. 32.4% (*p* = 0.78); T4: 13.3% vs. 46.7% vs. 40.0% (*p* = 0.12).

## Discussion

For many years, the high morbidity and mortality [7, 9, 10] and the uncertain survival advantage of P-Ar [6, 9] have discouraged surgeons from undertaking such major procedures. More recently, several groups have reported encouraging results [8, 9, 12, 34–37], the AJCC has removed the status “unresectable” from the definition of T4 [38], and NCCN guidelines included resection among the treatment options for LAPC following neoadjuvant treatments [39]. The growing interest in P-Ar is also shown by the description of newer approaches to deal with LAPC, such as arterial divestment following primary chemotherapy [40–42].

However, patient selection has been shown to be essential in improving results. Despite false-negative and false-positive results can occur [43], Ca 19.9 is the most widely used biomarker in pancreatic cancer and is currently a cornerstone in the selection of surgical candidates [18–20, 44].However, despite careful selection, outcomes of P-Ar for LAPC are sometimes frustrating even in patients with low pre-operative levels of Ca 19.9. In this manuscript, we have defined several prognostic factors that can help in a better selection of patients with LPAC undergoing P-Ar. No similar analysis is available in the medical literature. Molecular or genetic parameters [17, 45] are probably the best predictors of survival in all cancers, including PDAC, but these determinations are rarely performed in clinical practice and are unlikely to become available soon on a large scale.

In this study, we decided to use CSS to develop the prognostic score. OS is frequently used for this purpose, but it includes also deaths not directly related to cancer recurrence. DFS is also an excellent time-to-event endpoint in surgical oncology, but it can only be estimated from the time of surgery onward, thus missing the period of pre-operative therapy. Additionally, DFS does not take into account the event of cancer recurrence responding to additional oncologic treatments demonstrating a more favorable tumor biology worth to be captured in an oncologic prognostic score.

Based on CSS, we have identified eight prognostic factors, four of which had a negative impact and four a protective value. The negative prognostic factors were platelet count, Ca 15.3 level, Ca 125 level, and neutrophils-to-lymphocytes ratio. The protective factors were metabolic deterioration of diabetes, lymphocytes-to-monocytes ratio, platelets-to-lymphocytes ratio, and FOLFIRINOX-based neoadjuvant chemotherapy. Based on these eight factors, a prognostic score was created. Ca 19.9 did not appear to predict prognosis in this highly selected group of patients undergoing P-Ar because it was used to select patients for surgery, so that patients with high levels were not accepted.

The prognostic score identified three well-defined risk groups, marked by clear differences in median CSS (high-risk group: 16.05 months; IQR: 12.3–28.29), (intermediate-risk group: 24.77 months; IQR: 17.66-NA) (low-risk group: 39.01 months; IQR: 22.76–NA), mirrored by increased mortality rates due to cancer recurrence at 1, 2, and 3 years. While a median CSS time of 16 months could be probably achieved in several patients by state-of-the-art medical treatments alone, we cannot see how any combination of medical treatments and/or interventional procedures could result in a median CSS of 39 months and, more importantly, in a 5-year CSS of 46.0% and in a 8-year CSS of 30.6%.

The prognostic model could not be validated externally, because data on Ca 125 and Ca 15.3 levels were missed at other institutions. However, the prognostic model achieved a Harrell’s C-Index of 0.74, thus showing a good predictive value. Additionally, risk groups were found to match with pathology parameters known to predict CSS such as proportion of N2, lymph-node ratio, and logarithm odds of positive lymph nodes [13, 46, 47].

The prognostic model is based on eight variables: platelet count, Ca 15.3 level, Ca 125 level, neutrophils-to-lymphocytes ratio, lymphocytes-to-monocytes ratio, platelets-to-lymphocytes ratio, diabetes mellitus, and FOLFIRINOX-based neoadjuvant chemotherapy.

Experimental evidence supports the hypothesis that platelets contribute to cancer aggressiveness by means of multiple mechanisms [48]. Clinical data confirm that high platelet count has negative prognostic implications [49].Platelet-to-lymphocyte ratio is also a recognized prognostic marker in PDAC [50]. Interestingly, at least for the purpose of this discussion, recent studies have shown interactions between Ca 19.9 levels and both platelet count and platelet-to-lymphocyte ratio [51, 52]. Chen and co-workers have shown that the positive prognostic implications of low Ca19.9 levels are enhanced by low platelet counts and diminished by high platelet counts [51]. Sakamoto and co-workers showed similar interactions between Ca 19.9 levels and platelet-to-lymphocyte ratio [52].Thus, in the setting of purposefully low Ca 19.9 levels, such as in the current series, interactions with platelet levels and platelet-to-lymphocyte ratio could have further hidden the prognostic relevance of this tumor marker.

Regarding the other factors found to predict survival in this series, both neutrophil-to-lymphocyte ratio [53, 54] and lymphocyte-to-monocyte ratio [55] are well established prognostic factors in PDAC.

Ca 125 is emerging as a new marker of poor prognosis in PDAC [56–58]. Ca 125 is of immediately practical value in Lewis antigen-negative patients [43], and was found to be potentially superior to Ca 19.9 in predicting resectability in patients with high levels of serum bilirubin [59].

The prognostic value of Ca 15.3 in PDAC is instead a totally new piece of information. Ca 15.3 was shown to predict chemoresistance and early recurrence in breast cancer [60].

We also noted that metabolic deterioration of diabetes at the time of diagnosis was clearly associated with better survival (OR = 0.22). For the purpose of this analysis, metabolic deterioration of diabetes defines the condition in which patients with an established diagnosis of type 2 diabetes require the addition of insulin to oral antidiabetic therapy to maintain an acceptable metabolic control. While many patients with PDAC have concomitant diabetes, and there is a complex interplay between PDAC and diabetes, it is known that diabetes has prognostic relevance in PDAC [61]. Also some antidiabetic medications have prognostic implications in PDAC. Treatment with sulfonylureas, inducing hyperinsulinemia, was indeed associated with worse prognosis; while treatment with metformin, lowering insulin resistance, was associated with improved outcomes [62]. Additionally, metformin was shown to influence several cellular pathways involved in development and progression of PDAC [61] and to increase sensibility of pancreatic cancer cells to gemcitabine and 5-fluorouracil [62]. Our patients with improved survival had been under metformin therapy, before metabolic deterioration, and have continued to take metformin during pre-operative chemotherapy along with insulin supplementation.

Finally, there is little doubt that FOLFIRINOX-based neoadjuvant chemotherapy is beneficial in LAPC [63]. Actually, pre-operative chemotherapy is mandatory in all patients with LAPC before a P-Ar can even be considered. Our study shows that FOLFIRINOX should be preferred to gemcitabine-based regimens in patients undergoing P-Ar. However, low-grade evidence shows that in resectable and borderline-resectable pancreatic cancer, neoadjuvant chemotherapy with gemcitabine/nab-paclitaxel achieves survival outcomes similar to those of FOLFIRINOX. Considering that gemcitabine/nab-paclitaxel is better tolerated than FOLFIRINOX, this regimen could be conveniently employed in older patients with comorbidities [64–66].

This study provides some additional information; first, P-Ar remains associated with high morbidity and mortality rates. In our series, we have recorded a mortality rate of 10.3% at 90 days. This rate, although in keeping with several previous reports [7, 9, 10], still raises concerns on the safety of P-Ar. Our high mortality rate possibly reflects the high prevalence of resection and reconstruction of the SMA (38/77; 49.3%). This high mortality, however, could also reflect a learning curve effect. Indeed, as reported in other experiences, we have seen a decrease in mortality in recent years, with no additional deaths in the last 29 consecutive patients. So far, no study has addressed the issue of learning curve and surgeon/surgical team competence in these extended procedures, and no benchmark exists for P-Ar.

Second, it is noteworthy that in our series, over 20% of P-Ar were performed for tumor types other than PDAC, including 9% for malignant intraductal papillary mucinous neoplasms. Interestingly, no data on the outcomes P-Ar for non-PDAC tumors have been reported.

Third, 70% of resected arterial segments showed no actual tumor infiltration and nearly 80% of our patients had negative margins at 1 mm. These figures demonstrate that a radical procedure can be performed in properly selected patients with LAPC. These figures could also indicate that some arterial resections could have been spared, given that tumor infiltration was not confirmed at final pathology. To avoid this occurrence, some groups advocate the use of frozen section histology of peri-arterial tissues [67]. However, the reliability of this analysis is strongly limited by the high rate of false-negative results (33%), especially when biopsy samples are taken from tissues around the SMA [68]. Additionally, frozen section histology is proposed to abort resection in case of a positive result and to proceed further in case of a negative result, while divesting the artery. Adopting this policy, some arterial resections could be spared, but the surgeon should be prepared to accept a higher rate of margin positivity. Indeed, even in case of negative frozen histology it is unrealistic that cancer cells are > 1 mm from the periadvential plane, in patients with radiologic evidence of arterial involvement > 180°. Finally, in case of circumferential tumor growth around the vessel (360°), avoiding an arterial resection means that the “tumor cylinder” has to be breached, thus making the resection an overtly palliative procedure.

Fourth, resection of SMA was not associated with worse prognosis as compared to resection of celiac trunk/hepatic artery.

Fifth, nearly 70% of our patients could receive adjuvant chemotherapy, 39% received both pre- and post-operative chemotherapy, and 55% did not require dose reductions. These figures are similar to those recorded in patients with immediately resectable PDAC receiving upfront surgery and adjuvant chemotherapy [69], and challenge the concept that following P-Ar patients do not recover timely to receive post-operative medical treatments. However, more studies on recovery and quality of life after P-Ar are seriously needed.

This study has several limitations. First, despite prospective collection of data, the long study period carries the risk of time-dependent biases. Second, we miss an external validation cohort because of the rarity of P-Ar and difficulties in finding external series with assessment of Ca 15.3 and Ca 125 levels. Third, despite reporting on a relatively large number of P-Ar, the power of our study may not be sufficient to capture less obvious prognostic factors. As a result, some prognostic factors could be missed. Fourth, the overall management of cancer patients has improved over time. Although these improvements alone cannot probably explain the wide differences seen in median CSS times in the three risk groups, they could have had an impact. Finally, our patients are referred from all over the country. Socioeconomic, cultural, and geographical factors may have had an impact on the quality on non-surgical care delivered to these patients.

In conclusion, we have reported on the outcomes of P-Ar for LAPC. In patients with low Ca 19.9 levels, we have identified several, easily available, pre-operative prognostic factors that can be used to improve the selection of surgical candidates. An online calculator is available at www.survivalcalculator-lapdac-arterialresection.org. It is worth to note that we were able to identify two groups of patients with poor and good survival probability. Probably, patients in the high-risk group should not be offered surgery, while surgery should be recommended in the low-risk group. Patients in the intermediate-risk group should be managed on an individual basis.
